# Kynurenine metabolites predict survival in pulmonary arterial hypertension: A role for IL-6/IL-6Rα

**DOI:** 10.1038/s41598-022-15039-3

**Published:** 2022-07-19

**Authors:** Zongye Cai, Siyu Tian, Theo Klein, Ly Tu, Laurie W. Geenen, Thomas Koudstaal, Annemien E. van den Bosch, Yolanda B. de Rijke, Irwin K. M. Reiss, Eric Boersma, Claude van der Ley, Martijn Van Faassen, Ido Kema, Dirk J. Duncker, Karin A. Boomars, Karin Tran-Lundmark, Christophe Guignabert, Daphne Merkus

**Affiliations:** 1 Department of Cardiology, Erasmus MC, University Medical Center, PO Box 2040, 3000 CA Rotterdam, The Netherlands; 2 Department of Clinical Chemistry, Erasmus MC, University Medical Center, Rotterdam, The Netherlands; 3 INSERM UMR_S 999, Hôpital Marie Lannelongue, Le Plessis-Robinson, France; 4 School of Medicine, Université Paris-Saclay, Le Kremlin-Bicêtre, France; 5 Department of Pediatrics/Neonatology, Sophia Children’s Hospital, Erasmus MC, University Medical Center, Rotterdam, The Netherlands; 6 Department of Clinical Epidemiology, Erasmus MC, University Medical Center, Rotterdam, The Netherlands; 7 Laboratory Medicine, University Medical Center Groningen, University of Groningen, Groningen, The Netherlands; 8 Department of Pulmonary Medicine, Erasmus MC, University Medical Center, Rotterdam, The Netherlands; 9 Department of Experimental Medical Science, Lund University, Lund, Sweden; 10 Wallenberg Centre for Molecular Medicine, Lund University, Lund, Sweden; 11 Walter Brendel Center of Experimental Medicine (WBex), University Clinic Munich, LMU Munich, Munich, Germany; 12 German Center for Cardiovascular Research (DZHK), Partner Site Munich, Munich Heart Alliance (MHA), Munich, Germany; 13 Department of Cardiology, The Second Affiliated Hospital, Zhejiang University School of Medicine, Hangzhou, Zhejiang China

**Keywords:** Risk factors, Cardiovascular biology, Cardiovascular diseases, Cardiovascular biology, Biomarkers, Epidemiology, Translational research, Inflammation

## Abstract

Activation of the kynurenine pathway (KP) has been reported in patients with pulmonary arterial hypertension (PAH) undergoing PAH therapy. We aimed to determine KP-metabolism in treatment-naïve PAH patients, investigate its prognostic values, evaluate the effect of PAH therapy on KP-metabolites and identify cytokines responsible for altered KP-metabolism. KP-metabolite levels were determined in plasma from PAH patients (median follow-up 42 months) and in rats with monocrotaline- and Sugen/hypoxia-induced PH. Blood sampling of PAH patients was performed at the time of diagnosis, six months and one year after PAH therapy. KP activation with lower tryptophan, higher kynurenine (Kyn), 3-hydroxykynurenine (3-HK), quinolinic acid (QA), kynurenic acid (KA), and anthranilic acid was observed in treatment-naïve PAH patients compared with controls. A similar KP-metabolite profile was observed in monocrotaline, but not Sugen/hypoxia-induced PAH. Human lung primary cells (microvascular endothelial cells, pulmonary artery smooth muscle cells, and fibroblasts) were exposed to different cytokines in vitro. Following exposure to interleukin-6 (IL-6)/IL-6 receptor α (IL-6Rα) complex, all cell types exhibit a similar KP-metabolite profile as observed in PAH patients. PAH therapy partially normalized this profile in survivors after one year. Increased KP-metabolites correlated with higher pulmonary vascular resistance, shorter six-minute walking distance, and worse functional class. High levels of Kyn, 3-HK, QA, and KA measured at the latest time-point were associated with worse long-term survival. KP-metabolism was activated in treatment-naïve PAH patients, likely mediated through IL-6/IL-6Rα signaling. KP-metabolites predict response to PAH therapy and survival of PAH patients.

## Introduction

Pulmonary arterial hypertension (PAH) is characterized by an increase in pulmonary vascular resistance due to pulmonary vascular remodeling. This increases right ventricular afterload, eventually leading to progressive right heart failure^1^. There is currently no therapy reversing pulmonary vascular remodeling. Hence early identification of the disease as well as delineation of novel mechanisms contributing to pulmonary vascular remodeling are still of utmost importance to improve prognosis of patients with PAH.

**Figure 1:**
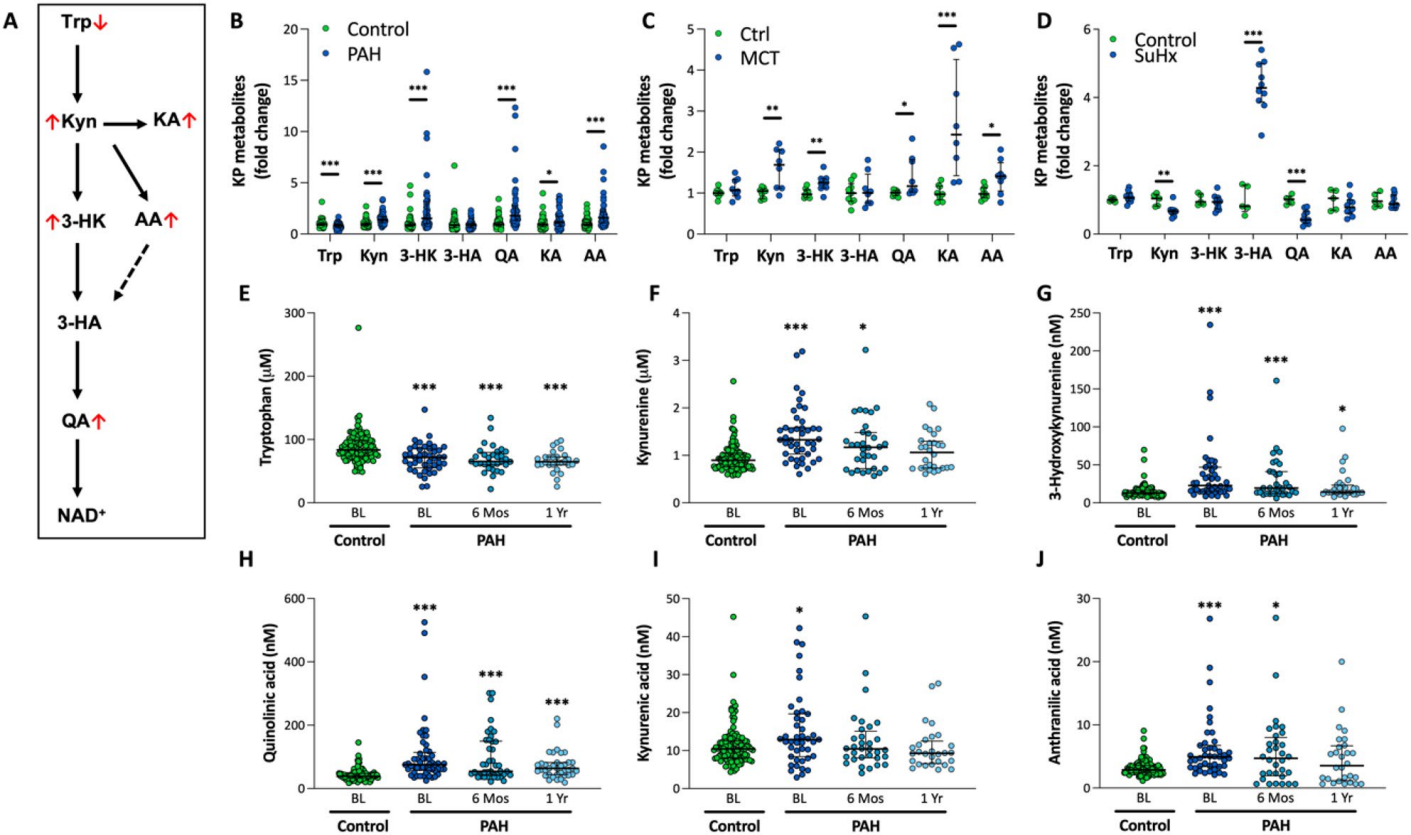
KP metabolite profile in PAH patients and two animal models of PH. (**A**) Scheme of de novo NAD^+^ synthesis through the kynurenine pathway with changes observed in PAH patients shown as red arrows. KP metabolite profiles in: (**B**) PAH patients at baseline (n = 43) vs Healthy controls (n = 111) in the controls), (**C**) MCT-PH rats (n = 8) vs Control rats (n = 9), (**D**) SuHx-PH rats (n = 10) vs Control rats (n = 5). A KP metabolite profile similar to PAH patients was only found in the MCT-PH rats which show a severe inflammatory phenotype, indicating the potential link between inflammation and KP metabolism in PAH. When compared to the controls (n = 111), (**E**) Tryptophan was significantly lower in PAH at baseline (n = 43) and after therapy (6Mos, n = 32; 1Yr, n = 28), (**F**,**J**) Kynurenine and anthranilic acid were significantly higher in PAH at baseline and 6 months after PAH therapy, (**H**,**I**) 3-hydroxykynurenine and quinolinic acid were significantly higher in PAH at baseline and after therapy, (**G**) Kynurenic acid was only higher in PAH at baseline. Data are presented as dot plots with median (IQR). *P < 0.05, **P < 0.01, ***P < 0.001 vs Controls, Mann–Whitney Test. *MCT* monocrotaline, *SuHx* Sugen plus hypoxia, *Trp* tryptophan, *Kyn* kynurenine, *3-HK* 3-hydroxykynurenine, *3-HA* 3-hydroxykynurenic acid, *QA* quinolinic acid, *KA* kynurenic acid, *AA* anthranilic acid, *NAD* nicotinamide adenine dinucleotide, *BL* baseline, *Mos* months, *Yr* year.

Recent studies have highlighted the pathophysiological importance of inflammation and mitochondrial dysfunction in the development and progression of PAH^2–4^. Nicotinamide adenine dinucleotide (NAD^+^) has been demonstrated to be an important modulator of inflammation and mitochondrial function^5–10^. NAD^+^ can be produced from vitamin B3 via the Preiss-Handler pathway and its derivate nicotinamide riboside via the salvage pathway^10^. Interestingly, NAD^+^ synthesis via the salvage pathway is enhanced in patients with advanced PAH as well as in rodent models of pulmonary hypertension (PH)^11^. A very important pathway that determines NAD^+^ levels is the de novo synthesis through the kynurenine pathway (KP). This de novo NAD^+^ synthesis through the KP starts with the conversion of essential amino acid tryptophan (Trp) into kynurenine (Kyn), by indoleamine 2, 3-dioxygenase, followed respectively by 3-hydroxykynurenine (3-HK), 3-hydroxykynurenic acid (3-HA), quinolinic acid (QA), and finally resulting in NAD^+^ formation^12,13^. There are branching points in the KP, as Kyn is also metabolized to kynurenic acid (KA) and anthranilic acid (AA) (Fig. 1A)^12,13^. Interestingly, a correlation between circulating Kyn, QA, and AA and pulmonary vascular resistance has been reported in PAH patients^14^. Similarly, increased Kyn levels were observed in other PAH cohorts^15,16^, and correlated with immune dysregulation and clinical outcome in a follow-up period of 6 months^16^. Together, these studies highlight the importance of the KP in PAH. However, PAH patients received PAH therapy in these studies and the KP-metabolite profile in treatment-naïve PAH patients as well as the effect of PAH therapy on this profile are currently unknown.

Therefore, we aimed to investigate: (1) KP-metabolite profile in treatment-naïve PAH patients as well as in two rat models of PAH, (2) the effects of PAH therapy on this profile in PAH patients, (3) the prognostic values of KP-metabolites during a long-term follow-up period. Given the potential correlation between KP-metabolism and immunity/ inflammation, we also investigated (4) whether cytokines and hypoxia are able to change the KP-metabolism in human lung cells.

## Methods

### Study population

The study protocol was approved by the Erasmus MC ethical committee (MEC-2011-392 and 2012-512), and written informed consent was obtained from all PAH patients and healthy volunteers. All procedures were performed in accordance with Declaration of Helsinki. Patients were not involved in the design of the study.

In this prospective observational cohort study, which encompassed forty-three consecutive treatment-naïve adult patients with PAH (mean pulmonary artery pressure ≥ 25 mmHg and pulmonary artery wedge pressure ≤ 15 mmHg) diagnosed by right heart catheterization between May 2012 and October 2016 at Erasmus MC were included. Treatment-naïve was defined as the absence of any history of treatment with approved target medications for PAH, i.e. prostacyclin, endothelin receptor antagonists, or phosphodiesterase type 5 inhibitors, and the diagnosis of PAH was in accordance with the definition at time of inclusion^17,18^. Patients that aged < 18 years, that were not treatment-naïve, had an incomplete diagnostic procedure, or were uncapable of signing informed consent were excluded. Thirty-nine PAH patients were prescribed PAH targeted therapies following diagnosis according to the latest guidelines^17,19^. PAH patients were prospectively followed till the 1st of January 2019. The primary outcome was defined as all-cause mortality and lung transplantation. Survival status was checked in the Municipal Personal Records database.

A control group consisting of 111 healthy volunteers was selected from the cohort in the study of using 2D speckle tracking echocardiography to evaluate the normal myocardial strain values in healthy subjects. The selected subjects had normal results on physical examination and electrocardiography (ECG), had no (prior) cardiovascular disease or cardiovascular risk factors (hypercholesterolemia, hypertension (blood pressure above 140/90 mmHg at the time of visit), or diabetes mellitus). More details about these PAH and control cohorts have been previously described^20,21^.

### Animal models of PH

Animal experiments were approved by the Ethics Committee of the Université Paris-Saclay (#11484) and carried out in accordance with the Guide for the Care and Use of Laboratory Animals adopted by the National Institute of Health and Medical Research (INSERM).and were performed in accordance with the ARRIVE guidelines. Plasma and lung tissue collected during previous experiments in two well-established rat models of severe PAH was used for this study^22^. Briefly, monocrotaline (MCT)-induced PH (MCT) was established in 4 weeks-old male Wistar rats (Janvier Labs, Saint Berthevin, France), with a single subcutaneous injection of MCT (40 mg/kg, Sigma-Aldrich, Saint-Quentin-Fallavier, France), and evaluated after 3 weeks (MCT-PH n = 15, controls n = 15). Sugen-hypoxia-induced PH (SuHx) was established in 4 weeks-old male Wistar rats (Janvier Labs, Saint Berthevin, France), by a single subcutaneous injection of SU5416 (20 mg/kg, Sigma-Aldrich, Saint-Quentin-Fallavier, France) combined with exposure to normobaric hypoxia for 3 weeks followed by normoxia for 5 weeks (SuHx-PH n = 10, controls n = 5).

### Human and rat EDTA-plasma samples

At the time of diagnosis (baseline), peripheral venous blood sampling was performed during diagnostic right heart catheterization for PAH patients, while blood sampling was performed at the time of visit for healthy volunteers. Blood sampling was also performed in surviving PAH patients 6 months (± 3 months, n = 32) and 1 year (± 3 months, n = 28) after PAH therapy. In the rats, blood sampling was performed under anesthesia (2% isoflurane) prior to excision of the heart and lungs.

### In vitro study of cultured human primary lung cells

The effects of cytokines and hypoxia on KP-metabolism were studied in 3 different types of human primary lung cells from healthy donors: microvascular endothelial cells (MVECs, CC-2527, Lonza) from 2 donors, pulmonary artery smooth muscle cells (PASMCs, CC-2581, Lonza) from 2 donors, and lung fibroblasts including human lung fibroblasts (CC-2512, Lonza) from 1 donor, and one MRC-5 lung fibroblast cell line (ATCC®CCL-171™). All cells were cultured in the corresponding medium kits. The passage of MVECs, PASMCs, and fibroblasts used in the final experiments were P8, P8, and P7, respectively. Hypoxia with 1% oxygen was achieved in a standard incubator with variable oxygen control (Thermo Fisher Scientific). The concentration of human recombinant cytokines (R&D systems) in the medium was 20 ng/mL for TNF-α (210-TA-020/CF), IL-6 (7270-IL-025/CF), IL-6/IL-6Rα (8954-SR/CF) and TGF-β1 (7754-BH-005/CF). MVECs were exposed to cytokines or hypoxia in basal medium with 0.5% FBS for 24 h prior to collection of medium. PASMCs and fibroblasts were starved with serum-free medium for 24 h and then exposed to cytokines or hypoxia in serum-free medium for 24 h before collection of medium. The medium was centrifuged at 2000×*g* for 20 min at 4 °C and the clear supernatant was collected and stored in aliquots at − 80 °C.

### Measurement of KP-metabolites

An in-house developed assay by ultra-performance liquid chromatography-tandem mass spectrometry (UPLC-MS/MS) was used to determine the KP-metabolite levels as previously described^23^, using 10 µL sample mixed with 10 µL isotopically labeled internal standard, including deuterated Trp, Kyn, 3-HK, 3-HA, QA, KA, and AA (Buchem BV, Apeldoorn, The Netherlands).

The measurement of NAD^+^ in rat lung tissue was performed with an NAD/NADH assay kit (Abcam #65348) according to the manufacturer’s instructions. Briefly, 15 mg frozen rat lung tissue was washed in cold PBS, homogenized with 400 μl of NAD/NADH extraction buffer, and centrifuged for 5 min at 4 °C. The supernatant was then collected for NAD/NADH measurement.

### Statistical analysis

Normality of continuous data was evaluated by Kolmogorov–Smirnov tests. Continuous variables are presented as mean ± standard deviation (SD) or median [interquartile range (IQR)], categoric variables as numbers (percentages). Unpaired t-test or Mann–Whitney test were used to compare differences in continuous variables (e.g. metabolite levels) between 2 groups (e.g. human PAH vs controls). Wilcoxon matched-pairs signed rank test was used to compare differences in KP-metabolites between two time points (baseline vs 6 months, and baseline vs 1 year) in PAH patients. Chi-square test was used to compare the difference in categoric variables (e.g. sex, NYHA) between 2 groups (e.g. survivors vs non-survivors). Spearman correlation coefficients were used to determine correlations between different KP-metabolites, and correlations between KP-metabolites and baseline characteristics. Logistic regression was conducted to determine whether KP-metabolites were independent predictors that distinguishing PAH patients from healthy controls. Comparisons of survival between groups were performed using the Kaplan–Meier estimator with Breslow-Wilcoxon test and log-rank test. Univariate and multivariate (Corrected for Age, Sex, PAH types (iPAH or APAH), and PAH therapy type (no therapy or mono or double or triple therapy)) Cox proportional hazard regression were used to assess associations between KP-metabolite levels and mortality in PAH patients. Statistical analyses were performed using IBM SPSS software (version 21.0.0.1). *P* < 0.05 was considered statistically significant.

### Ethics approval

The (human) study protocol was approved by the Erasmus MC ethical committee and all procedures were performed in accordance with the Declaration of Helsinki. Written informed consent was obtained from all PAH patients and healthy volunteers. All animal procedures were performed conform to the guidelines from Directive 2010/63/EU of the European Parliament on the protection of animals used for scientific purposes and approved by the respective animal welfare committees of the institute.

### Consent for publication

All authors have read and approved the final manuscript and consent for publication.

## Results

### Characteristics of the study population

Baseline characteristics of treatment-naïve PAH patients at the time of diagnosis and healthy controls are summarized in Table 1. During a median follow-up of 42 [interquartile range: 32–58] months, twelve PAH patients (Non-survivors) reached the primary endpoint, the causes included end-stage heart failure (n = 5), euthanasia because of end-stage cardiovascular and pulmonary disease (n = 2), lung transplantation (n = 1), progression of systemic sclerosis (n = 1), multi-organ failure (n = 1), sudden death presumed cerebral (n = 1), and malignancy (n = 1). Seven of them reached the endpoint within six months after diagnosis and hence had no follow-up measurements taken. Non-survivors were older, had higher heart rate and shorter 6-min walking distance than survivors (Table 1).

**Table 1:** Baseline characteristics of all PAH patients and healthy controls.

	Control	PAH		
		All	Survivors	Non-survivors
N	111	43	31	12
**Aetiology**				
iPAH, n (%)		15 (35)	14 (45)	1 (8)
CTD-PAH, n (%)		17 (40)	8 (26)	9 (75)
CHD-PAH, n (%)		11 (25)	9 (29)	2 (17)
**Clinical characteristics**				
Age, years old	43 ± 13	53 ± 17 **	49 ± 14	62 ± 19^†^
Sex, women n (%)	59 (53)	29 (67)	20 (65)	9 (75)
sBP, mmHg	123 [115–128]	122 [114–132]	123 [116–132]	115 [106–132]
HR, beats/min	68 [62–76]	78 [67–90] **	76 [63–87]	87 [76–99]^†^
BMI, kg/m^2^	23.8 ± 2.9	27.0 ± 6.1 ***	27.6 ± 6.6	25.2 ± 4.5
eGFR, mL/min/1.73m^2^	–	66.8 ± 18.4	68.9 ± 16.9	61.3 ± 21.8
hs-CRP, mg/L	–	3.1 [1.5–10.5]	3.1 [1.2–9.0]	3.4 [2.1–22.3]
NYHA, I:II:III:IV	–	1:13:23:6	1:11:16:3	0:2:7:3
6MWD, m	–	337 ± 153	377 ± 136	198 ± 133^††^
**Right heart catheterization**				
mPAP, mmHg	–	50.5 ± 16.1	50.5 ± 15.9	50.6 ± 17.3
PAWP, mmHg	–	11.8 ± 5.6	11.6 ± 6.0	12.2 ± 4.9
PVR, WU	–	7.1 [5.1–11.8]	7.9 [5.6–12.0]	6.4 [4.2–11.3]
CO, L/min	–	4.7 [3.9–5.5]	4.6 [3.9–5.5]	4.9 [3.9–6.5]
CI, L/min/m^2^	–	2.5 [2.2–3.3]	2.5 [2.2–3.2]	2.5 [2.0–3.6]
**PAH therapy types at time of censoring**				
No therapy, n (%)		4 (9.3)	2 (6.4)	2 (16.7)
Mono-therapy, n (%)		9 (20.9)	4 (12.9)	5 (41.6)
Dual-therapy, n (%)		13 (30.2)	11 (35.5)	2 (16.7)
Triple-therapy, n (%)		17 (39.5)	14 (45.2)	3 (25.0)

Data are presented as mean ± SD, median [IQR], or numbers (percentages).

*PAH* pulmonary arterial hypertension, *iPAH* idiopathic PAH, *CTD-PAH* connective tissue diseases associated PAH, *CHD-PAH* congenital heart diseases associated PAH, *sBP* systolic blood pressure, *HR* heart rate, *BMI* body mass index, *eGFR* estimated glomerular filtration rate, *hs-CRP* high-sensitivity C-reactive protein, *NYHA* New York Heart Association classification, *6MWD* 6-min walking distance, *mPAP* mean pulmonary arterial pressure, *PAWP* pulmonary arterial wedge pressure, *PVR* pulmonary vascular resistance, *CO* cardiac output, *CI* cardiac index.

**P < 0.01, ***P < 0.001 PAH versus control.

^†^*P* < 0.05, ^††^*P* < 0.01, survivors versus non-survivors. Unpaired T Test, Mann–Whitney U Test, and Chi-Square.

### KP-metabolite profile in PAH

At the time of diagnosis (baseline), Trp was significantly lower in treatment-naïve PAH patients compared to controls, while Kyn, 3-HK, QA, KA and AA were significantly higher in treatment-naïve PAH patients and no significant difference in 3-HA was found (Fig. 1B).

Binary logistic regression analyses showed that KP-metabolites significantly distinguished PAH patients from controls at baseline both in a univariate model and when corrected for age, sex, and body mass index (Table 2). Moreover, manual stepwise logistic regression analyses showed that including the whole panel of altered metabolites in the model predicted better than only one metabolite by significantly increasing the Chi-square of the model (Table 2).

**Table 2:** Prediction of PAH with each 1 μM decrease for Trp and 1 nM increase in other KP metabolites by binary logistic regression.

	Odds ratio [95% CI]	*P* value	Chi-Square	*P* value
**Univariable**				
Trp	1.047 [1.024–1.070]	< 0.001	21.083	< 0.001
Kyn	1.003 [1.002–1.004]	< 0.001	39.370	< 0.001
3-HK	1.097 [1.051–1.144]	< 0.001	38.390	< 0.001
QA	1.053 [1.032–1.075]	< 0.001	56.114	< 0.001
KA	1.076 [1.023–1.132]	0.005	8.842	0.003
AA	1.924 [1.453–2.550]	< 0.001	40.132	< 0.001
Whole panel			92.492	< 0.001
**Adjusted for age, sex and body mass index**				
Trp	1.036 [1.011–1.060]	< 0.001	35.551	< 0.001
Kyn	1.003 [1.002–1.005]	< 0.001	57.983	< 0.001
3-HK	1.089 [1.043–1.137]	< 0.001	53.224	< 0.001
QA	1.047 [1.026–1.069]	< 0.001	63.944	< 0.001
KA	1.090 [1.029–1.155]	0.003	35.368	< 0.001
AA	1.870 [1.398–2.502]	< 0.001	54.705	< 0.001
Whole panel			93.187	< 0.001

*Trp* tryptophan, *Kyn* kynurenine, *3-HK* 3-hydroxykynurenine, *QA* quinolinic acid, *KA* kynurenic acid, *AA* anthranilic acid.

**Figure 2:**
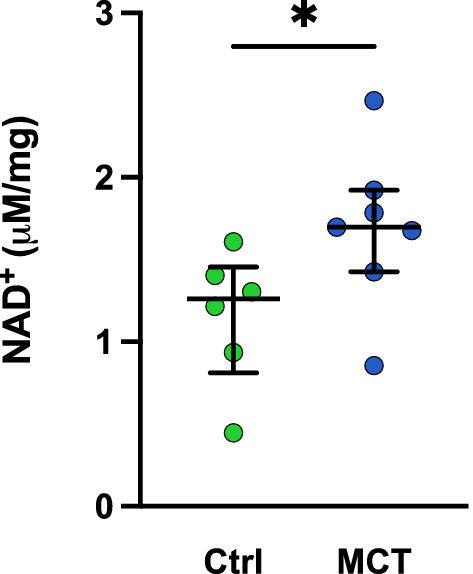
NAD^+^ levels in lung tissue are higher in rats with monocrotaline (MCT) induced PAH (n=6) as compared to Control rats (Ctrl, n=6). * *P* < 0.05.

MCT-PH rats showed a similar KP-metabolite profile as that observed in PAH patients (Fig. 1C), which was accompanied by an increase in NAD^+^ in the lungs from these rats (Fig. 2). Conversely, the KP-metabolite profile was different in SuHx-PH rats (Fig. 1D). As the MCT-PH rats show the most severe inflammatory phenotype, these results suggest a link between inflammation and KP-metabolism in PAH.

### IL-6/IL-6Rα contributed to the activation of KP-metabolism in vitro

**Figure 3:**
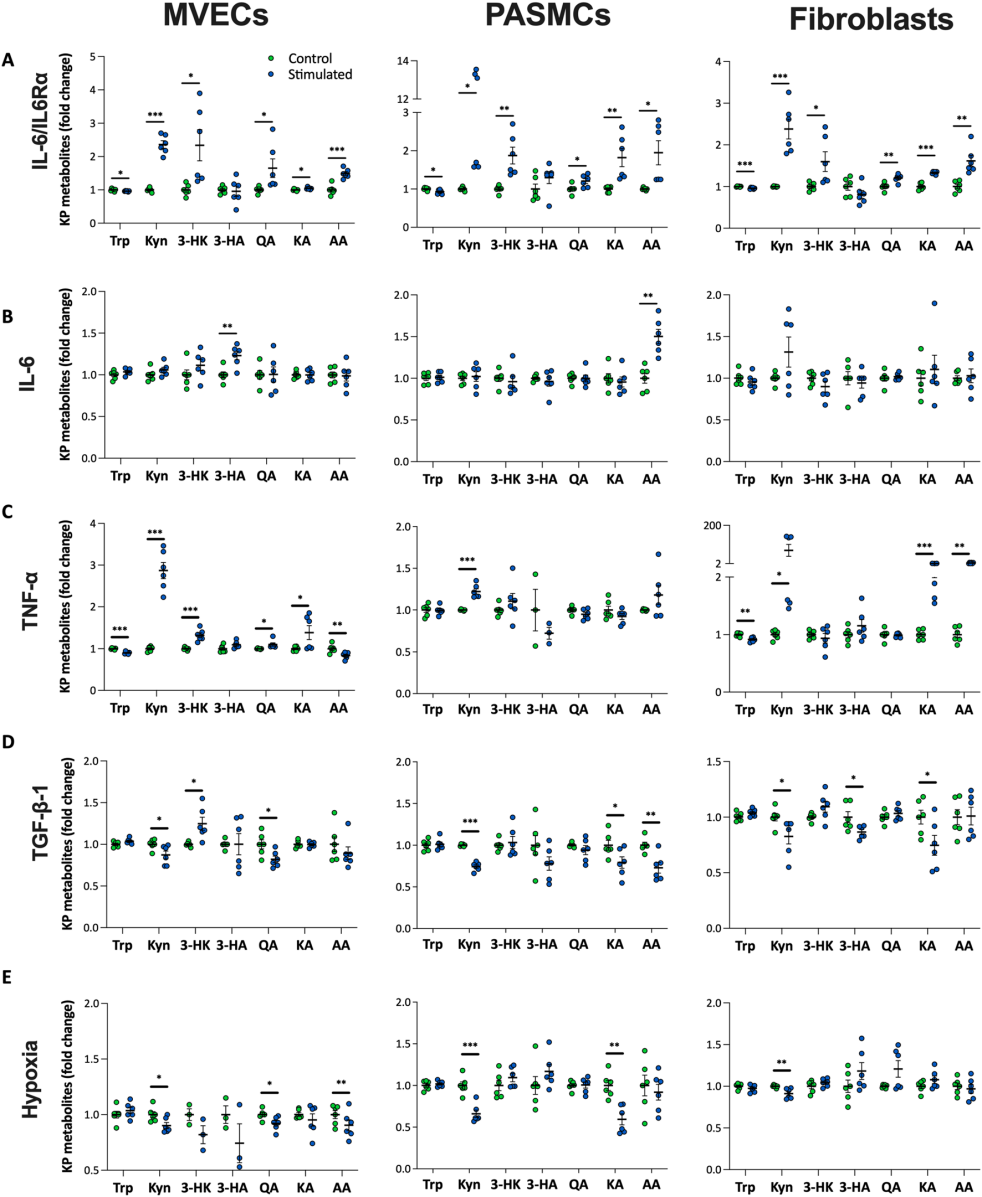
IL-6/IL-6Rα stimulation in vitro mimicked the abnormal KP metabolite profile observed in PAH patients. (**A**) Stimulation with IL-6/IL-6Rα in human lung MVECs (n = 6, 2 donors), PASMCs (n = 6, 2 donors), and lung fibroblasts (n = 6, 2 donors) for 24 h in vitro induced the abnormal phenotype of KP metabolism that was seen in PAH patients with lower Trp, higher Kyn, 3-HK, QA, KA, and AA, while unchanged 3-HA. Stimulation with IL-6 **(B),** TNF-α (**C**), TGF-β1 (**D**), or Hypoxia (1% O_2_, **E**) for 24 h in human lung MVECs, PASMCs, or fibroblasts in vitro resulted in different KP metabolite profiles. Data are presented as mean ± SEM, fold change to control, *P < 0.05, **P < 0.01, ***P < 0.001, Student T Test. *MVECs* microvascular endothelial cells, *PASMCs* pulmonary artery smooth muscle cells, *IL-6Rα* interleukin-6 receptor α, *IL-6* interleukin-6, *TNF-α* tumor necrosis factor α, *TGF-β1* transforming growth factor beta-1, *Trp* tryptophan, *Kyn* kynurenine, *3-HK* 3-hydroxy-kynurenine, *3-HA* 3-hydroxykynurenic acid, *QA* quinolinic acid, *KA* kynurenic acid, *AA* anthranilic acid.

Stimulation with IL-6/IL-6Rα complex induced KP activation and mimicked the KP-metabolite profile observed in PAH patients in all three cell types, while stimulation with IL-6 alone failed to induce a similar profile (Fig. 3A,B). Moreover, the cells responded differently to other cytokines and hypoxia failed to induce a KP profile similar to that seen in PAH patients (Fig. 3C–E). Taken together, these results indicate that inflammation, particularly activation of IL-6/IL-6Rα signaling contributed to the KP activation in PAH patients.

### Effects of PAH therapy on KP-metabolism

Following six months of PAH therapy, Trp was still significantly lower in PAH patients compared to controls, while only Kyn, 3-HK, QA, and AA were still significantly higher in PAH patients (Fig. 1E–J). After one year of PAH therapy, Trp was still significantly lower in PAH patients than in controls, while only 3-HK and QA were still significantly higher in PAH patients (Fig. 1E–J). These data suggest KP-metabolite profile partly normalized in PAH patients after PAH therapy.

**Figure 4:**
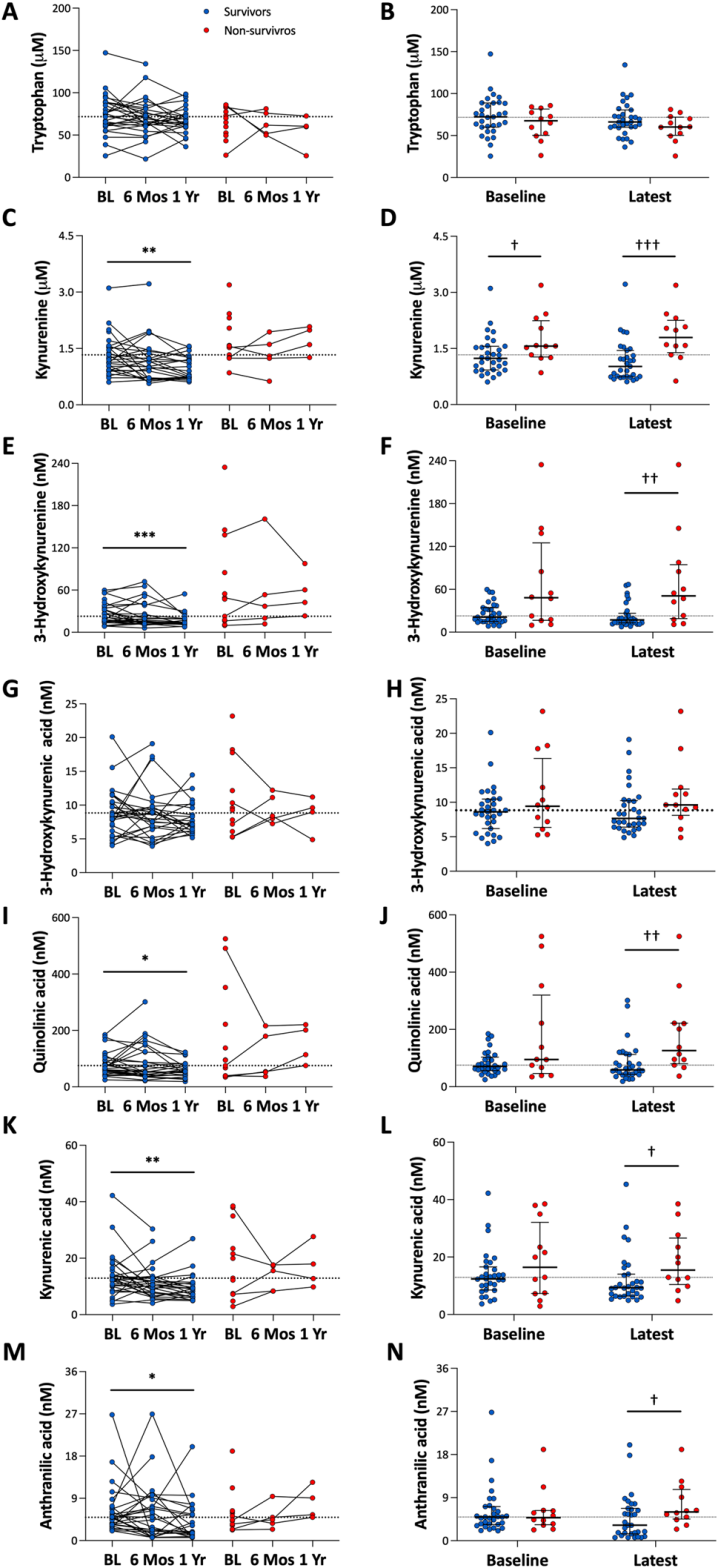
Treatment effects on KP metabolite levels in surviving vs non surviving PAH patients. A Mixed-effects model showed a decrease over time in Kyn (P = 0.010, C), 3-HK (P = 0.022, E), KA (P = 0.050, I), and with a trend toward significance for QA (P = 0.082, G).  Wilcoxon matched-pairs signed rank test showed that Kyn (*P* = 0.005, **C**), 3-HK (*P* < 0.001, **E**), QA (*P* = 0.013, **G**), KA (*P* = 0.007, **I**) and AA (*P* = 0.027, **K**) were significantly decreased in survivors after one year but not six months of PAH therapy. Moreover, at baseline, only Kyn was significantly higher in non-survivors when compared with survivors (**D**), while at the latest measurement after PAH therapy, Kyn (**D**), 3-HK (**F**), QA (**H**), KA (**J**) and AA (**L**) were all significantly higher in non-survivors when compared with survivors. Data are presented as dot plots with median (IQR), dash-lines indicate the baseline median value of KP metabolites in all PAH patients. *P* < 0.05, **P < 0.01, ***P < 0.001, Wilcoxon matched-pairs signed rank test. ^†^*P* < 0.05, ^††^*P* < 0.01, ^†††^*P* < 0.001, Mann–Whitney Test. *BL* baseline, *Mos* months, *Yr* year, *Trp* tryptophan, *Kyn* kynurenine, *3-HK* 3-hydroxy-kynurenine, *QA* quinolinic acid, *KA* kynurenic acid, *AA* anthranilic acid.

When baseline KP-metabolite levels were compared between survivors and non-survivors, only Kyn was significantly higher in non-survivors versus survivors (Fig. 4D). However, when comparing these levels at the latest measurement available, Kyn, 3-HK, QA, KA, and AA were all significantly higher in non-survivors compared with survivors (Fig. 4, right panels, time between baseline and latest measurement was 9.8 ± 3.8 months and 4.3 ± 5.5 months (mean ± SD) for survivors and non-survivors, respectively). In these survivors, Wilcoxon matched-pairs signed rank test showed that Kyn, 3-HK, QA, KA, and AA were significantly decreased after one year but not six months of PAH therapy, indicating that only long-term PAH therapy decreased KP-metabolite levels (Fig. 4, left panels).

These data indicate that KP-metabolites could be potential predictors of response to PAH therapy in survivors, and regular monitoring of KP-metabolites may be important to evaluate clinical status of PAH patients.

### Correlation of KP-metabolites and disease-severity

**Figure 5:**
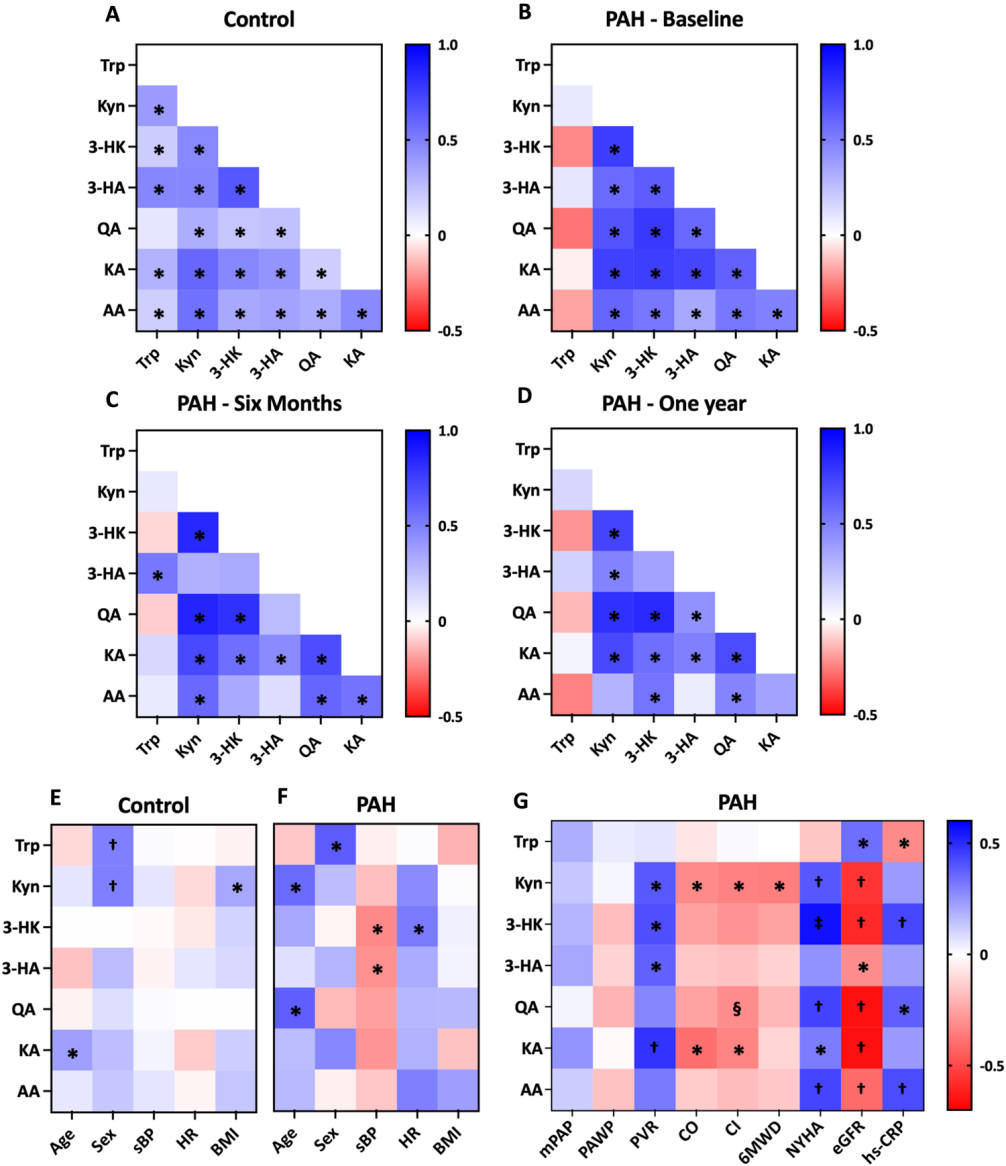
Correlation analysis. Correlation between KP metabolites in healthy controls and PAH patients. (**A**) KP metabolites correlated with each other in healthy controls except for Trp and QA. (**B**) At baseline, Trp lost its correlations with other metabolites, while other metabolites still correlated with each other in PAH patients. Such correlations were generally maintained after PAH therapy for six months (**C**) and one year (**D**). Correlations of KP metabolite levels with baseline characteristics. (**E**) In healthy controls, KA was associated with age, Trp and Kyn were associated with Sex, Kyn was associated with BMI. (**F**) In PAH patients, Kyn and QA were associated with age, Trp was associated with Sex, 3-HK and 3-HA were associated with sBP, 3-HK was associated with HR. (**G**) In PAH patients, Kyn, 3-HK, 3-HA, and KA were positively associated with PVR, Kyn, 3-HK, QA, KA, and AA were positively associated with NYHA, while Kyn was inversely associated with CO, CI and 6MWD, QA was inversely associated with CI, KA was inversely associated with CO and CI. Data are presented as rainbow heat map for Spearman coefficients, *P < 0.05, ^†^*P* < 0.01, ^‡^*P* < 0.001, ^§^*P* = 0.05, Spearman's Rank correlation coefficient. Sex: female = 0, male = 1; *Trp* tryptophan, *Kyn* kynurenine, *3-HK* 3-hydroxy-kynurenine, *3-HA* 3-hydroxykynurenic acid, *QA* quinolinic acid, *KA* kynurenic acid, *AA* anthranilic acid, *sBP* systolic blood pressure, *HR* heart rate, *BMI* body mass index, *eGFR* estimated glomerular filtration rate, *hs-CRP* high-sensitivity C-reactive protein, *mPAP* mean pulmonary arterial pressure, *PAWP* pulmonary arterial wedge pressure, *PVR* pulmonary vascular resistance, *CO* cardiac output, *CI* cardiac index, *6MWD* 6-min walking distance, *NYHA* New York Heart Association classification.

Significant correlations between different KP-metabolites were seen in healthy controls and PAH patients at baseline. In healthy controls, KP-metabolites correlated with each other, with one exception (lack of correlation between Trp and QA (Fig. 5A)). In PAH patients, Trp was not correlated with any other metabolite, while other metabolites still correlated with each other at baseline as well as after PAH therapy for six months and one year (Fig. 5B–D).

Significant correlations of KP-metabolite levels with baseline characteristics were observed both in healthy controls and PAH patients (Fig. 5E,F). Importantly, in PAH patients, higher levels of Kyn, 3-HK, 3-HA, and KA correlated with higher pulmonary vascular resistance, while higher levels of Kyn and KA correlated with reduced cardiac output and cardiac index, higher levels of QA correlated with a reduced cardiac index. Higher levels of Kyn, 3-HK, QA, KA, and AA correlated with worse functional class, higher levels of Kyn correlated with shorter 6-min walking distance (Fig. 5G). In addition, all KP-metabolites correlated with estimated glomerular filtration rate, and Trp, 3-HK, QA, and AA correlated with high-sensitivity C-reactive protein (Fig. 5G), further supporting the potential link between inflammation and KP-metabolism. C-reactive protein was not different between iPAH and aPAH subgroups.

### Survival analyses

**Figure 6:**
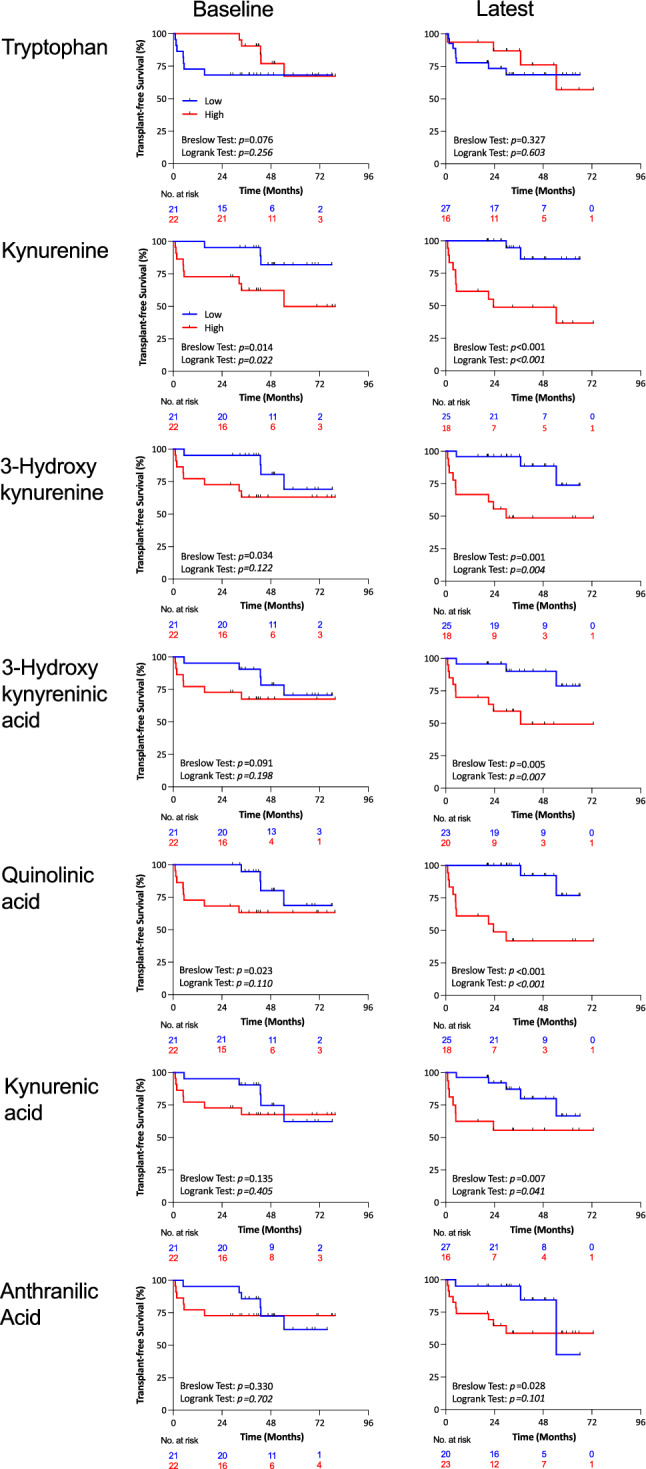
Survival analyses in PAH patients. PAH patients were stratified into 2 groups according to the median of baseline (left panels) or latest (right panels) KP metabolite levels. Using baseline metabolite levels, PAH patients with high levels of Kynurenine, 3-Hydroxykynurenine, or Quinolinic acid had worse transplant-free survival at short-term than patients with low levels (Breslow Test, *P* all < 0.05). Using the latest measurement, patients with high levels of Kynurenine, 3-Hydroxykynurenine, 3-Hydroxykynurenic acid, Quinolinic acid, or Kynurenic acid, had worse short- (Breslow Test, *P* all < 0.05) and long-term transplant-free survival (Log-rank Test, *P* all < 0.05) than patients with low levels, while patients with high anthranilic acid had worse short-term survival only.

PAH patients were stratified into two groups based on the median level of KP-metabolites measured at baseline. High levels of Kyn (> 1.328 μM), 3-HK (> 22.71 nM) or QA (> 75.23 nM) predicted worse early survival (Breslow Test), while high Kyn levels also predicted worse long-term survival (Log-rank Test, Fig. 6). However, there was no difference between patients with low and high levels of Trp, 3-HA, KA, or AA (Fig. 6).

Since survivors had lower levels of KP-metabolites at the latest measurement timepoint, which may be associated with a better response to PAH therapy, we compared the survival curves in PAH patients based on the latest available measurement. Again, high levels of Kyn, 3-HK, or QA predicted worse early survival, but also worse long-term survival (Fig. 6). In addition, patients with high levels of 3-HA (> 8.833 nM), KA (> 12.92 nM), or AA (> 4.944 nM) had worse early survival, and patients with high levels of 3-HA, or KA also had worse long-term survival (Fig. 6).

When considering KP-metabolites as continuous variables, each 1 nM increase in Kyn, 3-HK, QA, or KA was associated with an increased hazard ratio of death in both univariate and multivariate analyses, while 3-HA and AA were only associated with an increased hazard ratio in the multivariate model (Table 3). These results indicate that elevations in KP-metabolites are potential predictors of survival for PAH patients, with Kyn being the strongest prognostic biomarker.

**Table 3:** Cox proportional hazard analyses for death by 1 unit change in KP-metabolite levels in PAH patients.

	Baseline measurement		Latest measurement	
	Hazard ratio [95% CI]	*P* value	Hazard ratio [95% CI]	*P* value
Univariate*				
Trp	0.982 [0.954–1.010]	0.205	0.976 [0.945–1.007]	0.131
Kyn	1.001 [1.000–1.002]	0.012	1.001 [1.000–1.002]	0.001
3-HK	1.020 [1.009–1.030]	< 0.001	1.021 [1.010–1.032]	< 0.001
QA	1.006 [1.002–1.010]	0.001	1.007 [1.003–1.011]	0.001
KA	1.045 [0.996–1.096]	0.074	1.054 [1.007–1.104]	0.025
3-HA	1.119 [1.001–1.251]	0.048	1.114 [0.992–1.250]	0.067
AA	1.015 [0.906–1.138]	0.798	1.085 [0.987–1.193]	0.093
**Multivariate**^**#**^				
Trp	0.983 [0.954–1.014]	0.278	0.973 [0.937–1.011]	0.160
Kyn	1.001 [1.000–1.002]	0.022	1.001 [1.001–1.004]	< 0.001
3-HK	1.011 [1.003–1.020]	0.007	1.018 [1.007–1.029]	0.001
QA	1.006 [1.001–1.010]	0.010	1.006 [1.002–1.011]	0.006
KA	1.056 [0.998–1.117]	0.058	1.059 [1.004–1.116]	0.034
3-HA	1.124 [0.993–1.272]	0.064	1.166 [1.026–1.325]	0.019
AA	1.015 [0.902–1.142]	0.805	1.150 [1.010–1.310]	0.035

*Kyn* kynurenine, *3-HK* 3-hydroxy-kynurenine, *QA* quinolinic acid, *KA* kynurenic acid, *3-HA* 3-hydroxykynurenic acid, *AA* anthranilic acid.

*1 μM decrease for Trp, 1 nM increase for other metabolites.

^#^Corrected for Age, Sex, PAH types (iPAH or APAH), PAH therapy type (no therapy or mono or double or triple therapy).

## Discussion

The present study demonstrated that (1) KP-metabolism was activated in treatment-naïve PAH patients compared with healthy controls, (2) the KP-metabolite profile in MCT rats was similar to that in PAH patients and was accompanied by an increase in NAD^+^ levels in the lungs, (3) exposure of cultured MVECs, PASMCs, and fibroblasts to IL-6/IL-6Rα mimicked the KP-metabolite profile in PAH patients and therefore may contribute to KP activation in PAH patients, (4) PAH therapy normalized KP-metabolite levels in survivors, (5) KP-metabolites correlated with PAH severity at baseline and predicted mortality of PAH patients, particularly when using the latest measurement timepoint after PAH therapy.

This study is the first to measure the complete KP-metabolite profile in *treatment naïve* PAH patients and to repeat this measurement after six months and one year of PAH therapy. The results showed that PAH therapy normalizes levels of KP-metabolites in survivors after one year of therapy, which may explain why other PAH cohorts, with patients already undergoing PAH therapy, showed unchanged 3-HK, KA, and AA as compared to controls^14,16^. This suggests that PAH patients in these cohorts responded to PAH therapy. A limitation of our study is that only a single measurement was performed in the control cohort, so the effect of time on KP-metabolite profile in the control cohort could not be assessed.

Our results further demonstrated that KP-metabolites reflect disease severity in PAH patients, with higher levels of KP-metabolites correlating particularly with higher pulmonary vascular resistance, and/or worse NYHA class, and to a lesser extent with reduced cardiac index and shorter 6-min walking distance. Importantly, survival analysis showed that high levels of KP-metabolites (Kyn, 3-HK, QA, 3-HA, and KA) were strongly associated with worse mortality even after adjusting for age, sex, PAH type (iPAH or APAH) and PAH therapy type (no therapy or mono or double or triple therapy). Although the model may have been overfitted, as the number of events was relatively small, these results are in accordance with recent studies showing that higher levels of Kyn, 3-HK, and QA correlated with worse functional capacity and mortality in patients with heart failure^24,25^. Altogether, our data suggest that the KP-metabolite profile can be used as prognostic biomarker for PAH patients. However, detailed analysis of lung tissue obtained from PAH patients should be performed to investigate whether the expression of the enzymes involved in the production of the KP-metabolites is altered in the lungs. Such change in production is suggested by observations in lungs from rats with MCT induced PAH, that show an increase in Kyn and KA, consistent with the increased plasma levels observed in the present study.

In the present study, KP metabolites correlate with eGFR. This is in accordance with studies showing that KP metabolites are higher in patients with acute and chronic kidney disease^26^. However, KP metabolites did predict survival whereas eGFR did not a predict survival in our cohort (HR 0.979 [0.948–1.011, P = 0.201), although it has previously been shown that eGFR is a predictor of survival in PAH^27^. Because eGFR did not predict survival in this cohort, and the number of endpoints is relatively low, eGFR was not included in the multivariate analyses to prevent overfitting of the model.

Importantly, as changes in eGFR may also be linked to inflammation^28^, KP metabolites, eGFR and PAH may also be linked through inflammation. Indeed, immune dysregulation, inflammation and hypoxia are important factors that contribute to the development and progression of PAH^4^. The KP-metabolites 3-HK, QA and AA correlated with high-sensitivity C-reactive protein, a biomarker of inflammation and predictor of outcome in PAH^29^. As shown in Fig. 1B, these metabolites also show the largest increase as well as the largest variation between PAH patients. It is therefore likely that inflammation may cause differential alterations of KP enzymes in the lung. It is however, important to note that inflammation can also decrease eGFR. Hence, the association between KP-metabolites and eGFR may also represent a common consequence of inflammation. A link between KP-metabolites and inflammation is further supported by our observation that the MCT-PH rats, a model with an obvious inflammatory phenotype, showed a similar KP-metabolite profile as seen in PAH patients. Furthermore, it has been reported that inflammation in PAH patients is alleviated with PAH therapy in survivors^29^, which coincides with the normalization of KP-metabolite levels.

To further investigate the inflammatory mediator(s) underlying the KP activation, lung microvascular endothelial cells, smooth muscle cells and fibroblasts were exposed to a variety of inflammatory stimuli, such as IL-6, TNF-α, TGF-β and hypoxia. This in vitro study showed that particularly activation of IL-6/IL-6Rα signaling, which plays a prominent role in pulmonary vascular remodeling in PAH^30^, is an important modulator of KP-metabolism. Indeed, IL-6 was elevated in PAH-CTD as well as in a subgroup of iPAH patients in our cohort^31^. However, although 31 patients overlapped between this study and the present study, there was no correlation between KP metabolites and IL-6 (R^2^ < 0.05) except for a modest correlation between IL-6 and AA (R^2^ = 0.15).

Recently, PASMCs were found to be the local source of increased IL-6 in PAH^32^, while increased IL-6Rα has been shown in the MVECs^30^. Although the activation of IL-6/IL-6Rα pathway has been shown in both MCT and SuHx^30^, the KP-metabolite profile differed markedly between human PAH/MCT and SuHx. This difference may in part be explained by the observed reduction in Kyn levels in the different lung cell types in response to hypoxia. Nevertheless, treatment with tocilizumab, an IL-6R antagonist tested in the TRANSFORM-UK phase-II trial^33^, did not result in significant clinical improvement in PAH patients^34^ in spite of one case-report showing that tocilizumab improved symptoms in a patient with PAH associated with Castleman’s disease^35^. It would be interesting to measure the KP-metabolites prior to initiation and post treatment with tocilizumab to identify potential subgroups that might respond to tocilizumab and to further test a causal role of IL-6/IL-6Rα in KP activation.

Other inflammatory factors (IL-1b, IL-8, IL-10, CXCL9, CXCL13 and TGF-β) were measured within the same cohort (31 overlapping patients) and published in^31^. In addition to IL-6, IL-10, TGF-β, CXCL9 and CXCL13 were elevated in iPAH and/or aPAH patients. However, only CXCL9 correlated with all KP metabolites (R^2^ between 0.20 and 0.44) except Trp (R^2^ = 0.06). This correlation is in accordance with findings in COPD patients that CXCL9 correlates with Kyn/Trp ratio, as an index of KP activity^36^, and suggests that activation of inflammatory cells plays a role in KP-activation. However, this potential mechanistic link between CXCL9 and KP in PAH remains to be explored in future studies.

Although the KP is best known for its link with inflammation^6,13^, its metabolites have a wide variety of other functions. For example, Kyn is a vasodilator activating production of cAMP and cGMP/soluble guanylyl cyclase in the systemic^37,38^, coronary^38^, as well as pulmonary circulation^15^. Moreover, acute administration of *exogenous* Kyn reduced right ventricular systolic pressure in rodent models of PH^15^. If vasodilation induced by *endogenous* Kyn would be important in PAH, higher levels of Kyn should be associated with lower systemic blood pressure and/or lower pulmonary artery pressure. However, although PAH patients had higher levels of Kyn, Kyn correlated with higher PVR with no association with lower systemic or pulmonary artery pressure, and higher levels of Kyn correlated with higher pulmonary artery pressure in previous studies^14,15^. Therefore, a potential detrimental effect of KP activation that explains its association with worse prognosis in PAH may be mediated through other KP-metabolites.

NAD^+^, the end product of KP-metabolism, plays an essential role in regulation of mitochondrial function and has been implicated in aging/longevity^8^. Several studies suggest that NAD^+^ depletion is an important contributor to the pathogenesis of age-related diseases and cardiovascular diseases^8–10,39^, and raising NAD^+^ levels has been proposed as a promising therapeutic strategy for diseases such as obesity, renal diseases and heart failure^5,7,40,41^ by improving mitochondrial function, and prolonging survival of injured cells or apoptotic cells^5,42,43^. In contrast to these diseases, PAH is a disease in which mitochondrial remodeling^3^ is associated with excessive survival and proliferation of pulmonary vascular cells^4^. Thus, PAH seems to be associated with NAD^+^ abundancy that may contribute to pulmonary vascular remodeling. Indeed, nicotinamide phosphoribosyltransferase (NAMPT), the enzyme responsible for NAD^+^ synthesis via the salvage pathway, was increased in the circulation and lung of advanced PAH patients, and NAMPT inhibition has shown therapeutic effects in rodent models of PH by reversing pulmonary vascular remodeling^11^. In our study, higher levels of KP-metabolites also suggest an activation of de novo NAD^+^ synthesis in PAH patients, particularly since NAD^+^ levels were elevated in the lungs of MCT-PH rats. Unfortunately, there was no human lung tissue available to measure NAD^+^, and confirm rat data that activation of the KP is indeed associated with higher NAD^+^ levels. Nevertheless, available data suggest that KP activation might contribute to pulmonary vascular remodeling of PAH via de novo NAD^+^ synthesis. Hence, future studies should investigate whether inhibition of de novo NAD^+^ synthesis, via inhibition of KP activity, may provide a novel therapeutic target for PAH.

## Conclusion and clinical relevance

Activation of kynurenine pathway with increased levels of circulating KP-metabolites predict disease severity, response to PAH therapy, and survival in PAH patients. Exposure of lung microvascular endothelial cells, smooth muscle cells and fibroblasts to IL-6/IL-6Rα induced a KP-metabolite profile similar to that observed in PAH patients, suggesting that IL-6/IL-6Rα signaling likely contribute to KP activation in PAH patients. Whether KP activation contributes to pulmonary vascular remodeling via de novo NAD^+^ synthesis, and may provide a novel treatment strategy for PAH remains to be established.

## Supplementary Information

Supplementary Information.

## Data Availability

The datasets used and/or analysed during the current study are available as Supplementary Material.
